# Epidemiology, Secondary School Curricula, and Preparing the Next Generation for Global Citizenship

**DOI:** 10.2196/36006

**Published:** 2022-03-07

**Authors:** Charles E Basch, Corey H Basch

**Affiliations:** 1 Teachers College Columbia University New York, NY United States; 2 William Paterson University Wayne, NJ United States

**Keywords:** COVID-19, epidemiology, secondary school, global citizenship, emerging infectious disease, public health, teaching, student, high school education, population health

## Abstract

Because COVID-19 and other emerging infectious diseases are likely to play an increasingly important role in shaping American and global society in years to come, there is a need to prepare young people to make informed decisions in this changing global context. One way to do so is teaching and learning about basic principles of epidemiology in secondary schools. Improved understanding about the agent of infection, mechanisms of transmission, factors that increase or decrease susceptibility, place variation and environmental factors that facilitate or hinder transmission, reservoirs of infection (where the agent lives and multiplies), and when the disease is more or less likely to occur comprise the main facts about an infectious disease relevant to prevention and control. Improved understanding of these basic concepts could help future generations make informed decisions in a changing global context with emerging infectious diseases and a plethora of widely disseminated misinformation and disinformation. This viewpoint considers why learning about epidemiology in secondary school would benefit population health using COVID-19 as an illustration.

## Personal Choices in the Time of COVID-19

Controversies about personal choices and population health in the context of a global pandemic have been brought to the forefront as elected officials and citizens have taken sides regarding mandates for vaccination, mask use, and testing [1-5]. Concurrently, misinformation has been widely disseminated and has exacerbated public confusion as social media has changed the speed and reach of human communication [6-10]. COVID-19 and other emerging infectious diseases are likely to play an increasingly important role in shaping American and global society. This viewpoint considers why learning about epidemiology in secondary school would benefit population health using COVID-19 as an illustration.

As of the beginning of February 2022, there had been well over 5 million deaths worldwide from COVID-19 [11]. From the outset of the pandemic until the time of this writing (February 2022), the SARS-CoV-2 virus has disproportionately affected people based on age, race, and social determinants of health [12-15]. Additionally, COVID-19 has had universally negative global and domestic impact on education, the economy, physical and mental health, and family and community well-being [16-24]. COVID-19 is a global public health emergency [25].

As the novel coronavirus was spreading globally, another mass phenomenon was also occurring, namely, an *infodemic* [26,27]. Damage from misinformation (unintentionally incorrect or misleading information) and disinformation (intentionally incorrect or misleading information) is exacerbated by low levels of reading and health literacy, and a lack of understanding about disease transmission, prevention, and control [28-31]. It can be presumed that those who understand basic principles of epidemiology have an advantage in interpreting the chaos unfolding around them, making more informed decisions about reducing exposure and susceptibility, and contributing to individual, family, and community health [32,33].

The US Centers for Disease Control and Prevention (CDC) defines epidemiology as the study of the distribution and determinants of disease in human populations and their subgroups [34]. There are person (ie, host) factors, place factors, and time factors that influence the distribution and determinants of diseases and other populationwide phenomena (eg, crime and violence or addiction). Epidemiology is distinct from medicine because, although they are both grounded in science, the former concentrates on individual treatment, while the latter focuses on populationwide prevention and treatment [35,36]. As such, epidemiology is the fundamental science driving public health decision-making [34].

## Impetus for Change

Calls for including epidemiology in secondary school curricula are not new [37-42]. However, unlike any time in human history, COVID-19 revealed the challenge of educating the public as scientists scrambled to understand and create solutions in an environment where the people were routinely exposed to a plethora of misinformation and disinformation. There were several instances (eg, mask use and recommendations about the number of quarantine days required) when official government communications had to be revised or top governmental officials disagreed, which may have undermined public trust.

COVID-19 and other emerging infectious diseases are likely to play an increasingly important role in shaping American and global society in years to come. RNA viruses such as HIV, Chikungunya, and Dengue have been increasing over the last several decades [43-46]. This is also true for Lyme disease, which according to the CDC is affecting 20,000 to 30,000 people annually [47]. Coronaviruses in particular, of which there are hundreds, received attention from the severe acute respiratory syndrome (SARS) epidemic in Asia in 2003 [48] and Middle East respiratory syndrome (MERS) identification in the Arabian Peninsula in 2012 [49]. Mitigating the COVID-19 pandemic portends to change human interactions for the foreseeable future, and future generations need knowledge and health literacy skills to navigate inevitable epidemics in coming years.

There is strong justification for why learning about epidemiology in secondary schools is a sensible way to improve population health. First, to make informed decisions about reducing one’s exposure and susceptibility to SARS-CoV-2 and its variants, young people need to comprehend the causes of the disease, modes of transmission, factors that affect susceptibility, and *what is and is not known given current scientific understanding*. Second, the study of epidemiology affords many opportunities to complement core curriculum such as history, language, science, and mathematics in an interesting and applicable way. Third, a lack of knowledge leads people to be more vulnerable to fake news, enables manipulative efforts of unscrupulous companies attempting to profit from a public health emergency, and can result in behaviors that compromise one’s health and those around them. Fourth, greater understanding about the science of epidemiology may help people distinguish differing points of view rooted in politics. We believe all adolescents should learn about the basic principles of epidemiology to be engaged citizens relevant to disease prevention and health promotion in general and with respect to emerging infectious diseases and COVID-19 in particular.

## Basic Epidemiology of COVID-19

From the outset, we should be clear that our perspective on teaching about epidemiology is grounded in a health education lens. As such, the goal is focused on informed decision-making rather than particular behavior changes, with longer term aspirations related to quality of life and upward social mobility. There is a commitment to change by choice and to addressing the many social determinants of health that in too many cases constrain the ability to act on motivation. Another point to emphasize is that we focus on primary and secondary prevention because, despite historical investment in tertiary prevention (treatments after clinical disease has already occurred), primary and secondary prevention confer greater and more cost-effective benefits for population health.

Among the most basic facts when understanding any infectious disease is identifying the agent of infection, which for our purposes is SARS-CoV-2 and emerging variants [50]. There is also a need for basic understanding of mechanisms of transmission, which for COVID-19 include both direct and indirect possibilities. The mechanisms by which SARS-CoV-2 may be transmitted through direct contact would-be proximal contamination from someone coughing or sneezing directly on to another person while in close range [50]. During the early part of 2020, emphasis was placed on indirect transmission through fomites (ie, inanimate objects) [51], and less emphasis was placed on airborne aerosols and droplet transmission [50], though it became increasingly clear that the latter is responsible for the majority of propagated (person-to-person) transmission [50]. The implications here are for individuals to avoid densely populated and poorly ventilated indoor spaces, to social distance, and to use effective masks consistently and correctly when in poorly ventilated crowded spaces [50]. Another important basic fact is that humans infected with the disease are the major reservoir of infection [50]. It is also useful to understand the natural history of the disease, including the predisposing, presymptomatic, clinical, and convalescence stages. A centrally important fact is when an infected individual is most capable of transmitting the virus to a susceptible person, which in the case of SARS-CoV-2 can range from before symptoms appear to the ensuing days after. There are many important biological questions regarding how the virus is mutating and the extent to which variants are more pathogenic (contagious), virulent (proportion of severe and fatal cases), or resistant to vaccines [52].

### Person Factors

A key aspect of descriptive epidemiology includes describing the characteristics of people that increase or decrease the likelihood of disease occurrence. Person (or host) factors are traditionally classified into biological, physical, and social characteristics, but from a health education lens, behavioral, cognitive, and social/emotional characteristics are also important. The main biological factor related to COVID-19 is age, with older people at increased risk for more severe morbidity and mortality [53]. The main physical host factors are comorbidities (eg, cancer, chronic kidney disease, chronic lung diseases, certain neurological conditions, diabetes, and other diseases and treatments that may impede immune response) [54]. Social factors such as education and employment opportunities, income, and housing have a substantial effect on quality of life, longevity, and social mobility; this is exemplified in COVID-19 by the increased risk for people who have the lowest levels of income and education living in crowded, poorly ventilated spaces and having employment as essential workers [55-57]. According to the CDC, “the percent of Hispanic or Latino, non-Hispanic Black, and non-Hispanic American Indian or Alaska Native people who have died from COVID-19 is higher than the percent of these racial and ethnic groups among the total U.S. population” [58]. Person risk factors for COVID-19 of great interest are individual behaviors such as seeking and obtaining vaccination [51], social distancing [50], correct use of effective masks [50], and handwashing [50]. In turn, informed decision-making about these behaviors is influenced by cognitive factors such as awareness of controversial issues, up-to-date knowledge, and understanding that what is considered up-to-date has changed quickly in the COVID-19 pandemic. In turn, social/emotional factors such as fear, denial, disgust, and other feelings and emotions have been implicated in shaping individual choices as well [59-63].

### Place Factors

Place is centrally important to almost all diseases and to population health. Epidemiologists use mapping to describe where disease is more or less common. Inter- and intra-national comparisons, urban/suburban/rural variations, and localities with highest and lowest rates of disease provide clues about causation and where to prioritize. Once observing place variations, for example, through mapping, epidemiologists explore how the biological, physical, and social environmental factors may influence disease distribution. What environmental characteristics foster versus destroy pathogenic agents? Many bacteria and viral agents of infection cannot survive in or are washed away by soap and water; optimal hand hygiene is one the best ways to prevent transmission of many pathogens, including SARS-CoV-2 [64]. Consideration of the physical environment might call students’ attention to the importance of ventilation and how aerosols do or do not disperse within open air versus a crowded, poorly ventilated space [50]. As with most important population health issues, the social environment plays a dominant role in shaping morbidity and mortality outcomes. Social determinants have influenced risk of both infection and outcomes for COVID-19, with individuals with the lowest levels of income and education at highest risk [58]. Another example of how the social environment influences population health is investment in the scientific infrastructure that is needed to continue tracking and sequencing variants of SARS-CoV-2 [11]. In spring 2020, mapping showed that New York was the epicenter of COVID-19 in the United States.

The situation during March and April 2020 in New York City and New York State was dire and, sadly, was repeated in cities throughout the United States in the ensuing months, with health care systems becoming overwhelmed and the inability to manufacture and distribute personal protective equipment [65]. The pandemic has caused substantial negative effects on economies worldwide, especially those relying on population contact and movement (eg, transportation, tourism, food services, or event and entertainment) [16]. Politics and legislation provide many examples of contentious issues balancing the rights of individuals against harm to the community, and these issues play out in different ways in different states in the United States and globally. In places such as the United States where there is a large heterogeneous mix of cultural groups, challenges regarding what is and is not acceptable in terms of mask use, testing, containment, vaccination, and treatment seem inevitable.

### Time Factors

Another element of descriptive epidemiology that warrants consideration is time factors, such as secular trends, seasonal variation, cyclical patterns, and short-term spikes, as well as when an infected individual is most likely to transmit disease to others. The tracking system built by Johns Hopkins University continues to show where COVID-19 rates are increasing, decreasing, or stable in different localities and how well prevention efforts are working to reduce disease incidence. For example, data plotting the occurrence of new cases, hospitalizations, and mortality can help people understand the risk level in their community and influence highly consequential policies such as closing schools and businesses. Recognition that SARS-CoV-2 is most pathogenic (able to infect others) on the day before and in the days following the onset of symptoms is essential knowledge for informing guidelines for testing, isolation, quarantine, and community mitigation.

### Basic Metrics

Calculating primary metrics used in epidemiology such as incidence, prevalence, morbidity, and mortality rates only requires basic arithmetic. Likewise, understanding the ability of screening (testing) programs to distinguish people with and without the disease (sensitivity and specificity, respectively) can improve public understanding and decision-making. Another important health literacy skill is interpreting disease maps. These maps not only show where a disease is more or less common but also provide clues about the person and environmental factors that account for such variations. Herd immunity is another metric, which could be useful in helping individuals understand how and why their choices may cause harm to others.

### Prevention and Community Mitigation

Addressing any infectious disease epidemic generally requires multiple strategies, including reducing susceptibility and environmental exposures, disease surveillance, and eradication. For COVID-19, reducing susceptibility through vaccination and reducing environmental exposure by interrupting transmission are the most efficacious for community mitigation. These, in turn, require behaviors that rely on individuals’ decisions. Early detection, containment, and treatment are also essential elements of a comprehensive approach.

Reducing susceptibility through vaccination has been one of the most stunning examples of public health breakthroughs in the last century, eradicating smallpox and substantially reducing many infectious diseases that took a large toll on human populations globally. Development, emergency use authorization, and distribution of vaccines to prevent COVID-19 are a remarkable example of scientific progress. At the same time, there has been an active and influential movement against vaccination, which has undermined uptake levels. Students have a right to know the benefits and risks of various vaccinations to help them make informed decisions on a personal level.

A second key strategy for prevention and control of infectious diseases in general and COVID-19 in particular involves reducing environmental exposure by interrupting transmission. This is mainly accomplished through isolation (separating sick people from others), quarantine (separating those exposed to see if they become sick), social distancing, correct use of effective masks when in close proximity of others, and avoiding or taking extra precautions when in poorly ventilated enclosed spaces.

Although reducing populationwide susceptibility and environmental exposures are most effective to prevent disease from occurring, early detection, containment, and treatment are also essential elements of an effective overall public health response. There are a range of testing and contact tracing programs, each with its limitations. Testing and contact tracing has enabled identification and containment of hot spots and is an integral part of a more comprehensive strategy. The efficacy of alternative treatment approaches continue to be implemented and evaluated.

Improved understanding about the agent of infection, mechanisms of transmission, characteristics of people that increase or decrease susceptibility, environmental factors that facilitate or hinder transmission, reservoirs of infection (where the agent lives and multiplies), and when the disease is more or less likely to occur comprise the main facts about an infectious disease that can help contribute to prevention and control. To the extent that secondary school students are well informed about these topics, they will be in a better position to make informed decisions and contribute to prevention and community mitigation.

## Integrating Epidemiology into Secondary School Curriculum

The interdisciplinary orientation of epidemiology provides ample opportunities for practicing foundational skills in traditional academic subjects. Knowledge of the basic principles of epidemiology should be considered a cross-cutting topic that can help explain and reinforce academic skills across the curriculum. The following examples illustrate ways to integrate epidemiology into teaching and learning about history, language, science, and mathematics.

### History

The origins of epidemiology have been traced back to Hippocrates’ Humoral Theory of Disease around 400 BC as one of the first people to use rational versus supernatural thought to explain sickness and death. Galen of Pergamon expounded on Hippocrates theory in subsequent centuries along with Girolamo Fracastoro, Antonie van Leeuwenhoek, Ignaz Semmelweis, James Lind, Edward Jenner, John Snow, Louis Pasteur, and Robert Koch, among many others who contributed to the evolution of germ theory. More recent history tells of the remarkable discovery of antibiotics and vaccinations, which saved countless lives from historical scourges such as anthrax, bubonic plague, smallpox, cholera, measles, polio, and most recently COVID-19. There is an abundance of historical literature demonstrating how infectious diseases played a greater role in the outcome of war than military strategy [66,67]. Sadly, the role of how disease and famine continue to influence global populations provide examples of how studying peoples’ culture and history influences individual, family, and community health. One reason why epidemiology provides a fruitful lens through which to view history is that one of the main premises of epidemiology is that understanding factors that affect populations (and population health) requires recognition of a wide range of possible influences, which is clearly applicable when trying to understand and interpret historical records.

### Language

Not only does literature related to epidemiology provide opportunities for improving comprehension and vocabulary but it also provides examples of heroes throughout human history who devoted their lives to saving populations, how microbes have changed the course of war and geopolitical human history, and the extreme loss and human despair that disease has and continues to cause. Examples can be drawn from both fiction and nonfiction, for example, *Masque of the Red Death* (Edgar Allen Poe), *The End of October* (Henry Parsons), *Love in the Time of Cholera* (Gabriel García Márquez), *The Velveteen Rabbit* (Margery Williams), *The Plague* (Albert Camus), *The Ghost Map* (Steven Johnson), and short stories such as Stephen King’s *Nightsurf* or Boyle’s *After the Plague*. Lepore’s [68] article, What Our Contagion Fables Are Really About, reminds us that books can serve as a “salve and a consolation” especially in quarantine. Although the news and media throughout time covers the austere circumstances brought on by epidemics, Lepore [68] reminds us that “the existence of books, no matter how grim the tale, is itself a sign, evidence that humanity endures.”

### Science

The history of science is a phenomenal aspect of human evolution with respect to epidemiology. An example of a relevant narrative would be how humoral and miasma theories of disease fell by the wayside starting in the 1600s and culminating in greater acceptance of germ theory in the early 20th century. The concept of contagion and development of the microscope warrant study within a class on epidemiology, as would global epidemics throughout history such as the bubonic plague; cholera; smallpox; and more recently Zika, Ebola, MERS, and SARS, among other emerging infectious diseases that originated as zoonoses (transmissions from animals to humans). The 20th century topics might cover development and widespread use of antibiotics and vaccines for various diseases. New frontiers in the 21st century, being driven, in part, by epigenetics, are being fueled by billions invested to improve understanding about the biological, physical, and social interactions between populations (their genomes and oral and gut microbiomes) and the environment, as well as how interactions between microbial and human populations and the environment, including climate change, are shaping emerging infectious diseases and human health. Basic understanding of scientific methods could help individuals distinguish more from less credible messages and communications.

### Mathematics

At the heart of epidemiology is counting and making quantitative comparisons. In the 1600s John Graunt developed the Bills of Mortality in London, precursor to modern day vital statistics and global disease surveillance. These data are the basis for key aspects of domestic and global disease prevention and health promotion. Epidemiology can reinforce basic arithmetic skills by helping students learn how to calculate sensitivity, specificity, and predictive values of screening tests, and calculate relative and attributable risk and odds ratios. Basic understanding of public health metrics such as infant mortality, life expectancy, herd immunity, dependency ratios, crude, specific, and adjusted rates all provide opportunities for students to learn basic arithmetic skills while at the same time improving understanding about probability and risk, demography, and how risk and protective factors vary between population subgroups. One of the main proficiency areas in secondary school mathematics is quantitative comparisons, which is a key concept underlying descriptive epidemiology whereby disease rates of different subgroups are compared to generate hypotheses about possible causes.

## Discussion

What to include in secondary school curriculum is a value laden decision. In most countries, secondary school curricular decisions are handled centrally. In the United States, the conceptualization and design of curricular scope and sequence is decentralized at state and local community levels. Decentralization can be advantageous because it can help adapt education to local social and cultural contexts, and move away from standardized accountability metrics that only measure narrowly defined abilities. At the same time, decentralization created challenges for ensuring that students learn content and skills to reduce their exposure and susceptibility during the COVID-19 public health emergency [69].

We believe that improved understanding about epidemiology in general and about COVID-19 in particular can help people make informed decisions. This includes basic epidemiological facts about COVID-19: caused by the SARS-CoV-2 virus and variants; primarily spreads through aerosol and droplet infections but can also be spread through a variety of direct and indirect mechanisms; virus mainly lives and multiples in other people; virus cannot survive if exposed to soap and water; when presymptomatic and symptomatic pathogenicity (ability to infect others) is highest; and perhaps most importantly, that individuals’ decisions within an unequal and unfair set of constraints are the main determinants of becoming infected. What people know and are able to do plays a central role in risk reduction.

The emergence of SARS-CoV-2 and other infectious diseases result from human encroachment and destruction of the environment [70]. All indications sadly point to increased likelihood that zoonotic diseases will continue to evolve and infect humans. Now is an opportune time to rethink educational priorities to meet students’ needs as global citizens.

Epidemiology uses science-based evidence of disease patterns. Augmenting curriculum in history, language, science, and mathematics can help students learn useful health literacy skills while developing mastery in the subject. Basic understanding about epidemiology and public health requires awareness of the many ethical issues encountered in public health policy, laws, and practice. Of value to any young person’s education would be the ability to juxtapose individual freedoms versus public good, conflicting loyalties between funders and employers versus people served by programs, and the extent to which public health goals create agency and independence versus unsustainable dependency. Such education would align well with global citizenship.

Improved understanding about epidemiology is helpful for students because it will enable them to make more informed decisions during public health emergencies such as the COVID-19 pandemic and more generally for improving health literacy. Health literacy is predicated, in part, on searching for and evaluating the veracity of an increasingly growing amount of digital information. Distinguishing more from less credible information has become much more challenging in a context where digital communications instantly reach hundreds of millions of people and often contain misinformation and disinformation with respect to COVID-19 and a wide range of infectious diseases and other public health problems [71-76].

Epidemiology provides a useful framework and abundance of real-life examples for learning and practicing foundational skills related to history, language, science, and mathematics. In the current pandemic, students would benefit from improved understanding about how to calculate and reduce risks through practicing certain behaviors, and for more advanced students, simulations could be run illustrating the consequences for different proportions in a community behaving in different ways (eg, resulting in different levels of herd immunity).

The history of epidemiology shows how brilliant thinkers were able to view health problems from multiple perspectives, rebel against the dominant thought paradigm, and discover scientific truths that saved lives [77]. One of the most stunning public health findings in the last several decades is recognition that education is one of the most powerful predictors of well-being, social mobility, and longevity [78,79]. Now is an opportune time to reimagine education to help students make informed decisions in a rapidly changing global context.

## Abbreviations

CDC: Centers for Disease Control and Prevention
MERS: Middle East respiratory syndrome
SARS: severe acute respiratory syndrome
